# Carrion’s disease: an eradicable illness?

**DOI:** 10.1186/s40249-016-0197-7

**Published:** 2016-12-01

**Authors:** Cláudia Gomes, Maria J. Pons, Juana del Valle Mendoza, Joaquim Ruiz

**Affiliations:** 1 ISGlobal, Barcelona Ctr. Int. Health Res. (CRESIB), Hospital Clínic – Universitat de Barcelona, Barcelona, Spain; 2 Centro de Investigación e Innovación de la Facultad de Ciencias de la Salud de la Universidad Peruana de Ciencias Aplicadas, Avda. San Marcos cuadra 2, Chorrillos, Lima Peru

**Keywords:** *Bartonella bacilliformis*, Carrion’s disease, Oroya fever, Eradication, Neglected tropical diseases, Peru, Ecuador, Colombia, South America

## Abstract

Carrion’s disease is a neglected tropical disease caused by *Bartonella bacilliformis*, a vector-borne pathogen restricted to the Andean valleys of Peru, Ecuador and Colombia. Carrion’s disease is a biphasic illness; in the acute phase the case-fatality rate can be as high as 88 %, related to high parasitemia, arriving to almost all erythrocytes, and secondary bacterial infections close related with the development of transient immunosuppression in the earlier illness phases. In addition, there are an undefined number of asymptomatic carriers that are reservoirs of the etiological agent of Carrion’s disease in endemic areas, they make take into account due to they are the perpetuators of this disease. The actual scenario of Carrion’s disease, in which the illness is arriving to new areas, due to the expansion of the vector’s distribution, suggests that now may be a crucial time to design a strategy focusing on its elimination.

**Electronic supplementary material:**

The online version of this article (doi:10.1186/s40249-016-0197-7) contains supplementary material, which is available to authorized users.

## Multilingual abstracts

Please see Additional file 1 for translations of the abstract into the six official working languages of the United Nations.

## Background

In the last two centuries, the fight against infectious diseases has enormously progressed and the burden of a number of such diseases has dramatically been reduced, especially in developed countries. A series of milestones has marked this progress. The introductions of vaccines and antimicrobial agents in the 19th and 20th centuries, respectively, are considered to be the most crucial advances, with social developments, such as increasing education levels and improving nutritional status, access to potable water and improved sanitary environments also playing an invaluable role.

The eradication of smallpox and advances towards the eradication of poliomyelitis have been some of the major goals achieved by vaccination campaigns, and at present, the eradication of illnesses such as malaria, elephantiasis, teniasis, measles, mumps, rubella and yaws are red marked on the international health agenda, being performed strong efforts to advance towards this objective [1–3].

However, it is important to give attention to several other neglected diseases, such as Carrion’s disease. This infectious disease is caused by *Bartonella bacilliformis*, a vector-borne pathogen restricted to the Andean valleys of Peru, Ecuador and Colombia [4]. In Peru, the most affected country, endemic areas account for around 145 000 km^2^ of the total landmass, where more than 1.6 million inhabitants live [4]. Carrion’s disease is a biphasic illness, and has an acute and a chronic phase; in the acute phase the case-fatality rate can be as high as 88 %, in the event of no or delayed in treatment due to high parasitemia and secondary bacterial infections, which are associated to a *B. bacilliformis* induced transient immunosuppression. However, if the disease is correctly treated, the case-fatality rate decreases to around 10 % [4].

According to Peruvian national guidelines, the antibiotic treatments in the acute phase of the disease are chloramphenicol or ciprofloxacin, alone or combined with cephalosporins or aminoglycosides [5]. Meanwhile, rifampicin or azithromycin are proposed treatments in the chronic phase. Although *B. bacilliformis* is considered to be highly susceptible to all antibacterial agents except for quinolones [6, 7] and a recent study reported a 26 % resistance to ciprofloxacin and a 1 % resistance to chloramphenicol [8], in vitro studies have shown that *B. bacilliformis* may develop high resistance levels to almost all the aforementioned antibiotic agents [9, 10]. It is perhaps this phenomenon that contributes to the high case-fatality rate of Carrion’s disease.

The acute phase, so named Oroya fever, mostly affects previously non-exposed populations, such as children, as well as specific at-risk populations such as pregnant women due to the possibility that bacteria can be transferred through the placenta causing severe fetal complications including preterm birth, miscarriages, fetal death and neonatal Oroya fever, among others [4, 11–13]. Meanwhile, the chronic phase, which mainly affects previously exposed populations, is not life threating and is characterised by hemangioma-like nodules in the skin and mucous membranes known as ‘Peruvian wWarts’[4]. These lesions are mainly located in the extremities and head, possessing a variable morphology. Thus miliary, mulaire and nodule subdermal lesions have been described [4]. Asymptomatic infections are common in people from endemic areas. Although this phase has an undefined duration, a previous study reported the isolation of viable *B. bacilliformis* from blood samples of an Ecuadorian expatriate with acute splenomegaly and anemia who had been living out of endemic areas for three years [14]. Although definitive data about the real number of asymptomatic carriers, which act as perpetuators of the illness, are not available, Chamberlin et al. have reported that 45 % of inhabitants in endemic areas under 21 years of age showed previous exposure to the pathogen [15], using the indirect fluorescent antibody (IFA) method.

Thus, up until now no reservoir other than a human has been described. Several candidate reservoirs have been postulated in a number of studies during outbreaks of Carrion’s disease, from Euphorbiaceae to domestic and wild rodents. However, *B. bacilliformis* has never been identified in these candidate reservoirs [16]. In addition, studies on the vertical transmission of *B. bacilliformis* in *Lutzomyia verrucarum*, its main vector, have shown that high bacterial loads affect the viability of sandflies, hindering oviposition [17]. Despite control efforts, a resurgence of this infection has occurred [4] and has seemingly expanded to naive areas. This increase in infection may be related to the availability of diagnostic tools, vector expansion, climate change and human activities, such as the creation of new agricola areas or hydroelectric installations which may favours the life-cycle of *Lutzomyia* spp. or the creation of new roads and the increasing product trade, which may result in the accidental transfer of vectors to new areas, or due to the introduction of the illness into new areas by migrant populations from endemic regions. This suggests that now maybe a crucial time to design a strategy focusing on the elimination of Carrion’s disease.

## Main text

In the late years of the 20th century the actions towards eradication of different illness were increased. Thus in 1993 a series of infectious diseases were analysed to determine the feasibility of its eradication [18], and in 1997 a series of illness, including Chagas disease or leprosy were considered as target to be eliminated in 10 years period. Thus, being needed to establish in a firm manner the conditions needed to advance towards illness eradication, in 1997, Dahlem Workshop on the Eradication of Infectious Diseases was held in Berlin. One of the main things that came out of it was a series of criteria (Dahlem’s criteria) established to determine the feasibility of eliminating and eradicating an illness in the context of global health strategies [19].

Three specific points were considered to be the most relevant in the way towards diseases’ elimination: effectiveness of intervention, availability of sensitive diagnostic tools and human beings the only vertebrate reservoirs. Because Carrion’s disease meets all three criteria, it becomes evident that research efforts need to be strengthened to design and validate vaccine candidates to eradicate this disease. The high antibiotic susceptibility of *B. bacilliformis* suggests that mass treatment could lead to the elimination of the illness in some areas, and to a significant decrease of disease carriers in others. Additionally, this disease affect a well-delimited geographically area, being a key factor in the effectiveness of these actions. The development of molecular and immunological tools, including real-time polymerase chain reaction (PCR), enzyme-linked immunosorbent assays (ELISAs) and IFAs will allow for efficient diagnosis, however, the difficulties in their implementation in endemic areas, both related with the lack of adequate facilities and the need of training personnel, suggests the need for develop new rapid diagnostic tools to be directly used in rural areas. This point is extremely important in order to detect carriers after mass treatment or vaccination.

Economic and social factors should also be considered, as proposed at the Dahlem conference. Two points are of special concern: the need for consensus about action, where all organizations involved must act coordinated, including international support, and the perception of the illness as a pertinent public health issue.

The international support to fight against neglected tropical diseases (NTDs) has been strongly reinforced in the recent past. In 2012, the World Health Organization published a report highlighting the need to advance towards the control and eradication of NTDs, and alerting about the presence of several illness that have not been high on the global agenda [20]. Moreover, NTDs were one of the themes of the 2015 G7 summit in Germany. At the time, the G7 group made a firm commitment to advance the fight against these illnesses by supporting research in this area and investing in prevention and control efforts [21]. However, a disease that affects low- to middle-income countries and poor populations in remote areas and on non-touristic routes may not be perceived as a priority, and may therefore not attract sufficient interest and funding.

This lack of visibility is probably the biggest obstacle to eradicating Carrion’s disease. Furthermore, fragmented knowledge about the illness is also a challenge: its epidemiology is not yet fully understood, misdiagnosis occurs due to a lack of field-based diagnostic tools and there is a high variability in clinical symptoms [22]. The spreading of *L. verrucarum* to new areas is not sufficiently studied and there is a need for a proper surveillance system to discover potential secondary vectors, such as other species of *Lutzomyia* that are present in other South American regions, which may account for illness dissemination [12].

Despite adverse conditions, there are precedents in the fight against NTD in which the knowledge about has experimented substantial advances in short-time frame, providing of an armamentarium which may allow to advance towards control and eradication. The Ebola is an example, an ‘unknown’ disease that put the world on high alert. Thanks to political and social pressure, an effective vaccine (rVSV-ZEBOV) has been developed, which could signal the beginning of the end for massive Ebola outbreaks [23]. Presently, the window in which Carrion’s disease could potentially be eradicated might be about to close due to the aforementioned expansion of the illness to other areas. There is also risk of accidental introduction of vectors into remote areas, which can result in the expansion of the illness to faraway areas, similar to what Chikungunya caused in Southern Europe or Latin America [24, 25]. In the infectious diseases studies, pathogens that have been known during long time, in a sudden manner irrupt in the scientific agenda. An example is the Zika virus, a virus which is knows from 1953. At the moment of writing these lines, middle April 2016, we perform a PubMed research using the words “Zika virus” being found 458 results. Of these 334 (72.9 %) were from the present year. Maybe if this virus had been considered in a sound manner in the international research agenda prior to the last outbreak, the current situation will be different. It is time to become aware and act, before endemic diseases, like Carrion’s disease, expanding and will be a risk for global health.

## Conclusion

Currently, despite to be in slow expansion, Carrion’s disease, affects a well delimited geographical area, no reservoirs out of human has been described, antigenic candidates will may lead to vaccine or new diagnostic devices development have been proposed and antibiotics has a very good activity against causing microorganism. In summary, at present this is a potentially eradicable illness. Nonetheless, the insidious and slowly expanding Carrion’s disease continues to scourge the Andean valleys, being not one of the key scientific research interest areas globally and it is not a priority on international health agendas.

It may be that we wait to act at the time in which the illness arrive to touristic regions, or to big cities or when expanded out of Andean valleys. But at that time the windows-action will be closed.

## Additional file

Additional file 1: Multilingual abstract in the six official working languages of the United Nations. (PDF 226 kb)

## Abbreviations

IFA: Indirect fluorescent antibody
NTD: Neglected tropical disease
