# Supplementary material for: Carrion’s disease: an eradicable illness?
Source: Infect Dis Poverty. 2016 Dec 1;5:105. doi: 10.1186/s40249-016-0197-7 (PMC5131403; doi:10.1186/s40249-016-0197-7)
Supplement: Additional file 1: — Multilingual abstract in the six official working languages of the United Nations. (PDF 226 kb) [file 40249_2016_197_MOESM1_ESM.pdf]

Translation of the abstract into the six official working languages of the United Nations

داء كريون: هل يُمكن القضاء عليه؟

كلاوديا غوميس، ماريا ج. بونس، جوانا دال فالي مندوزا، جواكيم ريز

مُلخّص
يُعتبر داء كريون من الأمراض الاستوائية التي تمّ تجاهلها وهي من الأمراض الناتجة عن بكتيريا البرتونيلة العصوية (*Bartonella bacilliformis*)، وهذه البكتيريا ناقلة للمرض وتوجد في وديان كلّ من بيرو والإكوادور وكولومبيا. يتطوّر مرض كريون على مرحلتين: في المرحلة الحادّة، ترتفع نسبة حالات الموت لتصل إلى 88% والتي ترتبط بارتفاع نسبة وجود الطفيليات في الدم لتُصيب تقريبا جميع كريات الدم الحمراء، كما ترتبط بالالتهابات البكتيرية الثانوية المتعلقة بتطوّر الحدّ من المناعة المؤقت في المراحل المبكرة للمرض. كذلك، يوجد عدد غير مُحدّد من حاملي المرض دون وجود أعراض ويُعتبرون خزانات للعامل المسبب للمرض لداء الكريون في مناطق الوباء وينبغي أخذهم في الاعتبار لكونهم المسبب الرئيسي لهذا المرض. يقترح السيناريو الحالي المتعلق بمرض الكريون، وضع استراتيجية تسعى إلى القضاء على هذا المرض، فهذا هو الوقت المناسب للقيام بذلك حيث انتقل المرض إلى عدة أماكن بسبب انتشار ناقليه.

Translated from English version into Arabic by Zeineb TRABELSI, through

TRANSLATORS
WITHOUT BORDERS

巴尔通体病是一种可以消除的疾病吗？

Cláudia Gomes, Maria J. Pons, Juana del Valle Mendoza, Joaquim Ruiz

摘要
巴尔通体病，又称卡里翁病是由杆菌样巴尔通体（*Bartonella bacilliformis*）引起的一种被忽视热带病，秘鲁安第斯山脉的山谷地区、厄瓜多尔和哥伦比亚是该病的主要分布地区。该病是一种双相性的疾病，在急性期病死率可高达 88%，血液中含有大量病原体，几乎所有红细胞被侵犯。第二阶段为细菌感染期，与第一阶段短暂的免疫抑制密切相关。此外，还有未确定人数的无症状携带者作为该病流行区保虫宿主。事实上，由于媒介分布的扩张，巴尔通体病已经流传至其他地区，这也表明现在是设计策略消除该病的关键时期。

Translated from English version into Chinese by Xin-Yu Feng, edited by Pin Yang

CHINESE CENTER FOR DISEASE CONTROL AND PREVENTION
NATIONAL INSTITUTE OF PARASITIC DISEASES

Maladie de Carrión: une maladie éradicable?

Cláudia Gomes, Maria J. Pons, Juana del Valle Mendoza, Joaquim Ruiz

Résumé
La maladie de Carrión est une maladie tropicale négligée causée par la bactérie *Bartonella bacilliformis*, un agent pathogène vectoriel restreint aux vallées andines du Pérou, de l'Équateur et de la Colombie. La maladie de Carrión est une maladie biphasique. En phase aiguë, le taux de létalité peut s'élever à 88 % par rapport à la parasitémie élevée, étant donné l'affection de presque tous les érythrocytes et les infections bactériennes secondaires sont étroitement liées au développement de l'immunodépression transitoire au cours des phases précoces de la maladie. De plus, il existe un nombre indéfini de porteurs asymptomatiques qui servent de réservoirs de l'agent étiologique de la maladie de Carrión dans les zones endémiques et dont il convient de tenir compte, étant donné le fait qu'il sont à l'origine de la maladie. Le scénario actuel de la maladie de Carrión impliquant son arrivée dans de nouvelles régions suite à l'expansion de la répartition du vecteur suggère qu'il pourrait désormais s'avérer crucial d'établir une stratégie visant à son élimination.

Translated from English version into French by eric ragu, through

TRANSLATORS
WITHOUT BORDERS

Болезнь Карриона: искоренимая болезнь?

Cláudia Gomes, Maria J. Pons, Juana del Valle Mendoza, Joaquim Ruiz

Резюме

Бартонеллёз(болезнь Карриона) -- забытая тропическая болезнь, которую вызывают бартонеллы -- патоген переносимого вектора, органиченный на Андской долине в Перу, в Эквадоре и в Колумбии. Бартонеллёз является двухфазной болезнью, в острой стадии показатель смертности может достичь до 88%, это связано с тем, что паразитемия уже почти дошла до всех эритроцитов. И закрытие второй бактериальной инфекции имеет отношение с развитием кратковременной иммуносупрессии в более раннем этапе заболевания. Кроме того, существуют бессимптомные носители (количество неизвестно), они резервуары этиологического агента бартонеллёза в районах эндемии. Их обязательно учитывать, потому что они вечный сохранитель этой болезни. Ревльный сценарий, в котором болезнь доходит до новых зон из-за экспансией вектора, указывает, что сейчас важнее найти подход для элиминации болезни.

Translated from English version into Russian by Liang Ma, through

TRANSLATORS
WITHOUT BORDERS

Enfermedad de Carrión: ¿una enfermedad erradicable?

Cláudia Gomes, Maria J. Pons, Juana del Valle Mendoza, Joaquim Ruiz

Resumen

La enfermedad de Carrión es una enfermedad tropical desatendida producida por *Bartonella bacilliformis*, un patógeno transmitido por vectores que se restringe a los valles andinos de Perú, Ecuador y Colombia. La enfermedad de Carrión es una enfermedad bifásica. En la fase aguda la tasa de mortalidad puede alcanzar hasta el 88%, asociada con elevada parasitemia, que ataca a casi todos los eritrocitos, e infecciones bacterianas secundarias íntimamente asociadas con el desarrollo de inmunosupresión transitoria en las fases más tempranas de la enfermedad. Asimismo, existe una cantidad indefinida de portadores asintomáticos que son reservorios del agente etiológico de la enfermedad de Carrión en las zonas endémicas, a tener en cuenta ya que pueden ser quienes perpetúan la enfermedad. El escenario actual de la enfermedad de Carrión, en la que la enfermedad logra llegar a nuevas áreas, debido a la expansión de la distribución del vector, sugiere que este es un momento crucial para el diseño de una estrategia que se enfoque en su eliminación.

Translated from English version into Spanish by Maria Alejandra Aguada, through

TRANSLATORS
WITHOUT BORDERS
